# The Promotion of Erythropoiesis via the Regulation of Reactive Oxygen Species by Lactic Acid

**DOI:** 10.1038/srep38105

**Published:** 2017-02-06

**Authors:** Shun-Tao Luo, Dong-Mei Zhang, Qing Qin, Lian Lu, Min Luo, Fu-Chun Guo, Hua-Shan Shi, Li Jiang, Bin Shao, Meng Li, Han-Shuo Yang, Yu-Quan Wei

**Affiliations:** 1 State Key Laboratory of Biotherapy and Cancer Center, West China Hospital, Sichuan University, and Collaborative Innovation Center for Biotherapy, Chengdu, 610064, China; 2 Center of Reproductive Medicine, Department of Gynecology and Obstetrics, State Key Laboratory of Biotherapy, West China Second Hospital, Sichuan University, and Collaborative Innovation Center for Biotherapy, Chengdu, China; 3 Department of Oncology, Chengdu Shang Jin Nan Fu Hospital, Chengdu, Sichuan 610041, China; 4 State Key Laboratory of Biotherapy and Cancer Center, West China Hospital, Sichuan University, and Collaborative Innovation Center for Biotherapy, and Head and Neck Oncology Department of Cancer Center, West China Hospital, Chengdu, 610064, China; 5 West China Hospital, West China Medical School, Sichuan University, Chengdu, 610064, China

## Abstract

The simultaneous increases in blood lactic acid and erythrocytes after intense exercise could suggest a link between lactate and the erythropoiesis. However, the effects of lactic acid on erythropoiesis remain to be elucidated. Here, we utilized a mouse model to determine the role of lactic acid in this process in parallel with studies using leukaemic K562 cells. Treatment of K562 cells *in vitro* with lactic acid increased the mRNA and protein expression of haemoglobin genes and the frequency of GPA^+^ cells. Also, increases in haematocrit and CD71^−^/Ter119^+^ erythroid cells were observed in lactic acid-treated mice, which showed a physiological increase in blood lactate. Mouse bone marrow CD34^+^/CD117^−^ cells showed an increase in erythroid burst-forming units after stimulation with lactic acid *in vitro*. Furthermore, lactic acid increased the intracellular reactive oxygen species (ROS) content in bone marrow and in K562 cells. Erythroid differentiation induced in Haematopoietic Stem Cells (HSCs) and K562 cells by lactic acid was abolished by reducing ROS levels with SOD or 2-mercaptoethanol, which suggests that ROS is a critical regulator of this process. These findings provide a better understanding of the role of lactic acid in cellular metabolism and physiological functions.

Erythrocytes function to transport oxygen to tissues and are essential for the survival of all vertebrate animals. Erythropoiesis is a complex multistep developmental process that mainly occurs in the bone marrow during adulthood in humans, including multipotent hematopoietic stem cells (HSCs) differentiate, proliferate, and finally mature into red blood cells^1^,^2^,^3^. This process is initiated by the differentiation of HSCs into earliest erythroid progenitors: burst-forming-unit erythroid (BFU-E) cells, and then colony-forming-unit erythroid (CFU-E) cells. Subsequently, proerythroblasts produce basophilic, polychromatic, and orthochromatic erythroblasts. Orthochromatic erythroblasts finally differentiate to reticulocytes and subsequently become mature erythrocytes. The commitment and differentiation of HSCs responds to cellular metabolism and some bioactive substances *in vivo*, such as iron ions, short-chain fatty acids, amino acid and multiple cytokines^4^,^5^,^6^,^7^. Previous studies have shown that inhibition of mitochondrial fatty acid oxidation leads to symmetric commitment of HSC daughter cells and loss of HSCs maintenance^4^,^5^. Glycolysis has been shown to block differentiation of embryonic stem cells via promoting histone acetylation^8^. Research on amino acid metabolism has revealed that glutamine catabolism promotes histone/DNA demethylation and maintains pluripotency of stem cells via producing a high level of intracellular α–ketoglutarate (αKG)^7^. In addition, the commitment of HSCs to an erythroid lineage requires glutamine metabolism and glutamine-dependent de novo nucleotide biosynthesis. Blocking this pathway could divert HSCs towards cells with myelomonocytic fates^9^. An increased uptake of neutral essential amino acids (NEAAs) is required for erythropoiesis and inadequate NEAA uptake results in a specific reduction in haemoglobin production^10^.

Lactic acid is a small molecule metabolic product that is produced during anaerobic glycolysis in mammalian cells. Blood lactate concentration increases under physiological anoxic conditions, such as during intense exercise and high altitude hypoxia. Bone marrow provides a hypoxic niche, in which resident HSCs rely heavily on glycolysis and generate more lactic acid than other normal tissues^11^,^12^,^13^. Lactic acid also are vastly increased in diseases such as cancer, obesity, hypertension, and type 2 diabetes^14^,^15^,^16^. Tumour cells enhance lactic acid secretion by altering their glucose metabolism in hypoxic microenvironment. Lactic acid increases synchronously with erythropoiesis in some physiologically and pathologically hypoxic conditions, such as during intense exercise, long periods at high altitudes^17^, and in cancer patients with various types of tumours^18^,^19^. The synchronous increase in erythrocytes and lactic acid level indicates that lactic acid may have an effect on erythropoiesis. However, it remains unclear whether lactic acid has direct effects on erythropoiesis, although we have known the indirect relationship between lactic acid and erythropoiesis – lactic acid enhances the incorporation of iron ions into red blood cells in polycythaemic mice^20^.

In this study, we demonstrate that lactic acid directly promotes the erythroid differentiation *in vitro* and *in vivo*. Additionally, we clarify the role of ROS and their relationship to lactic acid and the specific process of erythropoiesis. Our data demonstrate that lactic acid promoting erythroid differentiation by intracellular ROS and Jak2-STAT5 pathway. These results suggest the pivotal role of lactic acid in physiological erythropoiesis.

## Results

### Lactic acid promotes K562 erythroid differentiation *in vitro*

**Figure 1:**
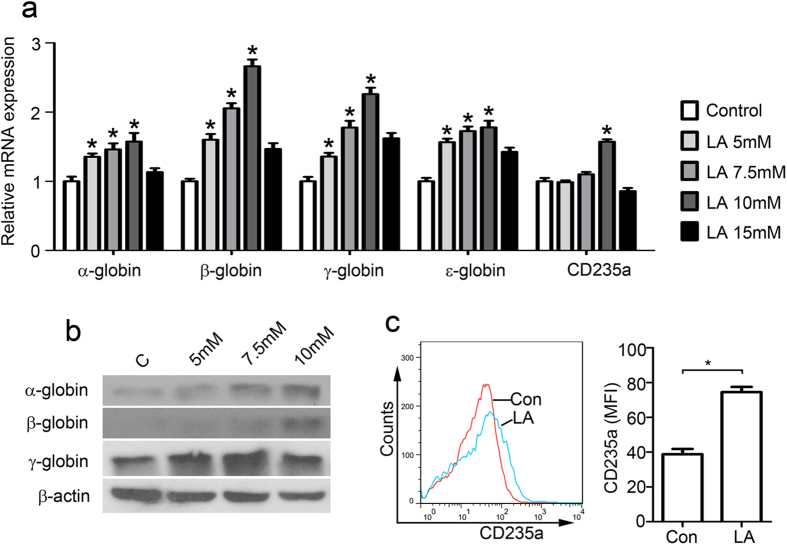
Lactic acid induces erythroid differentiation of K562 cells. (**a**) Haemoglobin and CD235a mRNA expression levels in K562 cells 72 hours after 10-mM lactic acid treatment. mRNA levels were normalized to the expression of β-actin mRNA. *P < 0.05 vs. their respective controls. (**b**) Haemoglobin expression in K562 cells at 72 hours after treatment with 5-, 7.5- or 10-mM lactic acid. (**c**) Flow cytometry demonstrates that CD235a expression in the K562 cells increased 3 days after lactic acid treatment. Mean fluorescence intensity (MFI) analyses are shown. *P < 0.05.

Human leukemic K562 cells are able to undergo erythroid differentiation *in vitro* when cultured with various kinds of inducers, and has been used as a useful *in vitro* model system to study erythroid and megakaryocytic differentiation^21^,^22^,^23^. Therefore, we investigate the effect of lactic acid on erythroid cell differentiation by utilizing K562 cells *in vitro*. Haemoglobin expression was detected using quantitative real-time PCR and immunoblotting on day 3 after lactic acid treatment. The mRNA levels of α-, β-, γ and ε-haemoglobin were significantly increased (1.57×, 2.66×, 2.26× and 1.77×, respectively) at 72 h after a 10 mM lactic acid treatment (Fig. 1a). However, lactic acid at 15 mM appeared to inhibit this increase in haemoglobin compared with the effect of the 10 mM treatment but still resulted in higher levels of β-, γ- and ε-haemoglobin than in the control group. Similarly, lactic acid increased the protein expression of α-, β-, and γ- haemoglobin at 72 h, suggesting the erythroid differentiation for K562 cells (Fig. 1b).

CD235a (Glycophorin A, GPA) is a marker of erythroid cell lineage. CD235a RNA levels increased significantly 72 h after 10-mM lactic acid treatment (Fig. 1a). Flow cytometry results revealed that compared with the control group, K562 cells treated with lactic acid exhibited higher CD235a. The mean fluorescence intensity (MFI) of the LA group increased by almost 2-fold compared with the normal group (Fig. 1c). These data indicated that erythroid differentiation of K562 is enhanced by lactic acid.

### Lactic acid promotes erythroid differentiation of bone marrow cells *in vitro*

**Figure 2:**
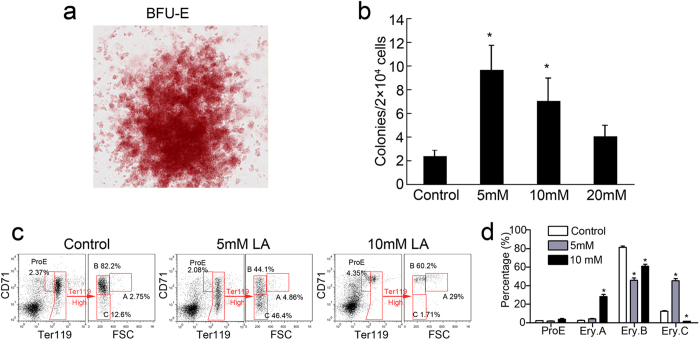
Lactic acid induces erythroid differentiation of BM cells. (**a**) Micrographs of BFU-Es derived from 10-day co-cultures of CD34^+^/CD117^−^ BM cells with lactic acid. (**b**) Enhanced the colony-formation activity of the CD34^+^/CD117^−^ BM cells 10 days after lactic acid treatment. The data represent the mean ± S.E.M. of three dishes. *P < 0.05 vs. the control. (**c**) Representative flow cytometric profiles of BM cells cultured in complete RPMI 1640 medium for 48 hours with lactic acid. (d) Frequencies of the populations are summarised in panel c. The data are reported as the mean ± S.E.M. (n = 3). *P < 0.05 vs. the control.

To further confirm the effect of lactic acid on erythroid differentiation, mouse CD34^+^/CD117^−^ cells from bone marrow (BM) were cultured and analysed *in vitro* with/without lactic acid treatment. After a 10-day incubation with lactic acid, the number of erythroid burst-forming units (BFU-Es) increased significantly in the CD34^+^/CD117^−^ BM cells treated with 5- or 10-mM lactic acid (Fig. 2a,b). However, a higher concentration of lactic acid (20 mM) eliminated the capacity of BFU-E promotion (Fig. 2b). A representative image of the BFU-E results is shown in Fig. 2a.

As previously described^24^, four erythroblast subsets were identified via the forward scatter (FSC) parameter, CD71 and Ter119: ProE (proerythroblasts), Ery.A (baso erythroblasts), Ery.B (late baso and poly erythroblasts) and Ery.C (ortho erythroblasts and reticulocytes) (Fig. 2c). Flow cytometric analysis showed an increase in the Ery.C population in the 5-mM lactic acid group at day 2 compared with the level in the normal group. The frequencies of the Ery.C population increased by approximately 3× (Fig. 2c,d). However, the frequencies of the Ery.C population in the 10-mM group decreased markedly, suggesting high doses of lactic acid may be toxic to BM cells. So, we detected the apoptosis of lactate-treated BM cells stained with PI and annexin V by using flow cytometry. The results showed a small increased annexinV^+^PI^−^ and annexinV^+^PI^+^ populations in the 10-mM lactate-treated group but not in the 5-mM lactate-treated group (Supplementary Figure 1). These results suggest that suitable lactic acid concentrations promote not only the generation of erythroid BFU-E progenitors but also the maturation of erythroid cells, whereas high doses of lactic acid may be toxic to normal BM cells but still can induce the erythropoiesis (Fig. 2b).

### Lactic acid promotes bone marrow erythropoiesis *in vivo*

**Figure 3:**
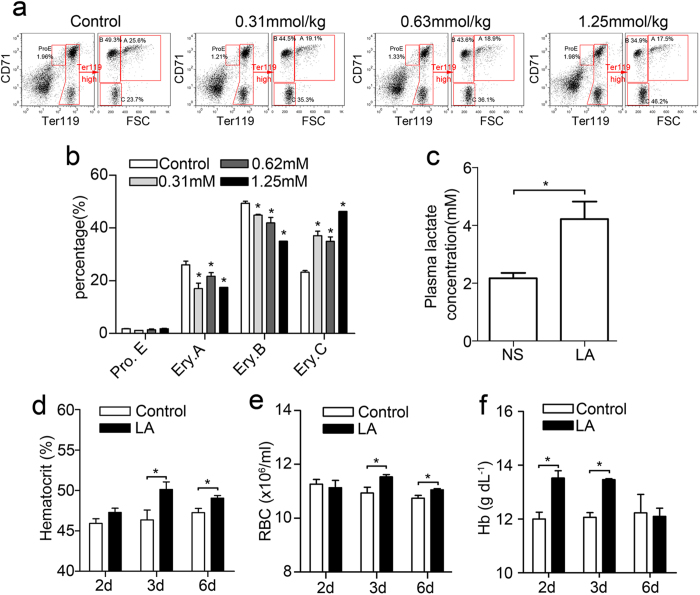
Bone marrow erythropoiesis promoted by physiological concentrations of lactic acid *in vivo*. (**a**) Representative flow cytometric profiles of BM cells from lactic acid-treated or normal mice at 48 hours. (**b**) Summary of the percentage of the populations shown in panel a. The data represent the mean ± S.E.M. of mice (n = 3). *P < 0.05 vs. their respective controls. (**c**) The plasma lactate levels 10 minutes after intraperitoneal injection of 1.25 mmol/kg lactic acid. The data represent the mean ± S.E.M. *P < 0.05. (**d**–**f**) The haematocrit, red blood cell counts and haemoglobin levels in the blood 2, 3 and 6 days after NS or 1.25 mmol/kg lactate infusion. The data represent the mean ± S.E.M. *P < 0.05 vs. their respective controls.

To examine the *in vivo* role of lactic acid in erythropoiesis as a regulator of the bone marrow microenvironment, four groups of mice were intraperitoneally (i.p.) injected with different doses of lactic acid. The frequency of erythroblasts in the BM was measured via FACS 48 hours following lactic acid administration (Fig. 3a). In the 1.25 mmol/kg LA-induced group, the most mature Ery.C population nearly doubled (Fig. 3b), whereas the Ery.B and Ery.A populations both declined by 0.3-fold. In groups stimulated with 0.31 mmol/kg or 0.63 mmol/kg lactic acid, the Ery.C population increased by approximately 1.5-fold, whereas the Ery.B and Ery.A populations slightly declined compared with the normal group. These results indicate that lactic acid promotes mature erythrocyte production in the BM by inducing the differentiation of basophilic and polychromatic erythroblasts into mature erythrocytes and that this effect was related to the concentration of lactic acid present.

To determine the concentration of lactate in the blood after the injections, we measured the lactate concentration 10 minutes after the injections, at which the injected lactate reaches its peak^25^. The result showed that plasma lactic acid increased to approximately 4 mM after the 1.25 mM/kg lactic acid injection (Fig. 3c), which is comparable to the levels observed in mice after exercise^26^,^27^. Therefore, the injection of 1.25 mM/kg lactic acid could reflect a physiologically relevant increase in lactic acid in mice.

To further detect the effect of lactate on erythropoiesis, we examined the haematocrit (HCT), red blood cell (RBC) numbers and haemoglobin (Hb) levels of peripheral blood at day 2, 3 and 6 after lactic acid treatment. The results showed that lactic acid-treated mice had higher HCT levels and RBC counts at day 3 and day 6 than control mice (Fig. 3d,e), and increased haemoglobin levels at 2 and 3 days after lactic acid treatment (Fig. 3f). Importantly, lactic acid treatment induced the significant increase of circulating Epo at 24 and 48 hours (Supplementary Figure 2, **P** < **0.05**). These results together indicated that lactic acid promotes bone marrow erythropoiesis *in vivo*.

### Generation of ROS via lactic acid treatment

**Figure 4:**
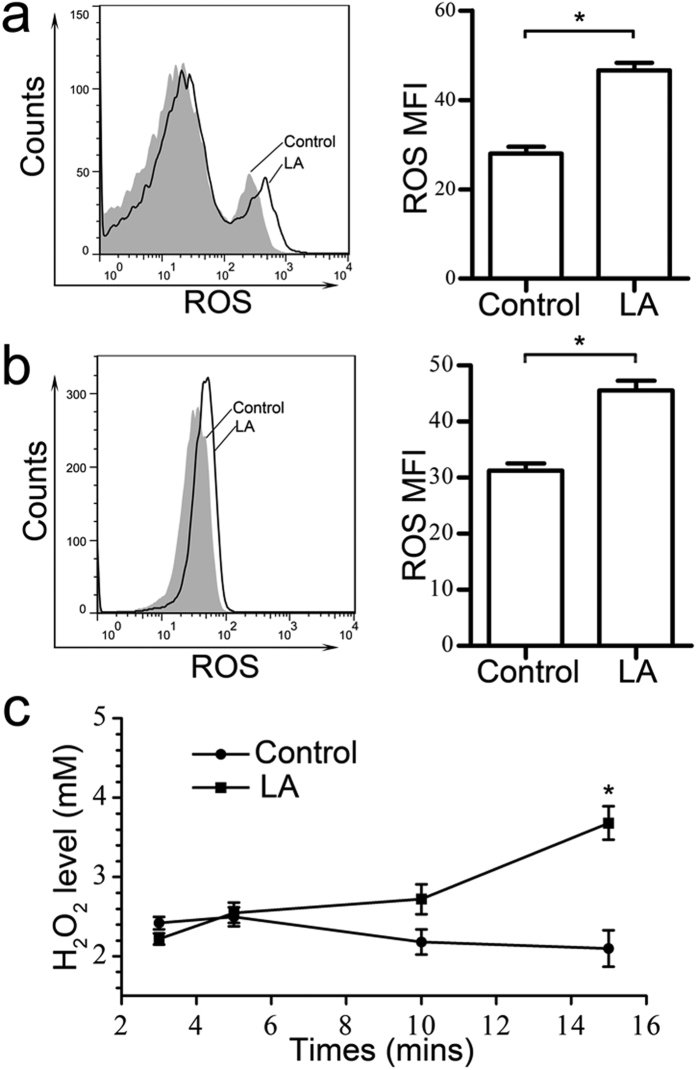
Increased ROS production in the BM cells and K562 cells treated with lactic acid *in vitro*. BM cells (**a**) and K562 cells (**b**) were treated with lactic acid for 6 hours and then pre-loaded with 10 μM DCFH-DA. DCF fluorescence was measured via flow cytometry. The black curves indicate lactic acid-treated K562 cells or BM cells, and the grey curves indicate untreated cells. MFI analyses are shown to the right. The data represent the mean ± S.E.M. (n = 3). *P < 0.05. (**c**) Enhanced production of H_2_O_2_ was observed in the K562 cells 15 minutes after the 10-mM lactic acid treatment. The data represent the mean ± S.E.M. *P < 0.05.

The mechanism for promoting erythropoiesis via lactic acid *in vivo* may include increased erythropoietin produced by the kidney; however, no erythropoietin production was detected via ELISA or western blotting in HSCs or in K562 cells cultured *in vitro*. Consequently, other mechanism must be involved in lactic acid-induced erythroid differentiation *in vitro*. Previous results have demonstrated that ROS may affect erythropoiesis^28^ and that lactic acid in particular alters the cellular oxidative status, inducing high levels of ROS in the L6 cells^29^. Thus, we hypothesized that lactic acid promotes erythropoiesis in HSCs and K562 cells by regulating cellular ROS levels. Measurements of cellular ROS levels in BM cells using the DCFH-DA probe detected via flow cytometry showed increased ROS 6 hours following 5-mM lactic acid treatment. The MFI of the ROS increased from 27 to 46 in lactate-treated cells (Fig. 4a). Similar results were obtained in K562 cells, in which 10-mM lactic acid caused a significant increase in ROS levels. The ROS MFI increased by 1.5-fold after lactic acid treatment compared with the normal group (Fig. 4b). We also detected an increase in cellular H_2_O_2_ levels less than 15 minutes after treatment using a previously reported method^29^. The H_2_O_2_ levels in the K562 cells began to increase at 10 minutes and showed a 1.76-fold increase at 15 minutes after lactic acid treatment (Fig. 4c).

### Lactic acid promotes erythroid differentiation in a ROS-dependent manner

**Figure 5:**
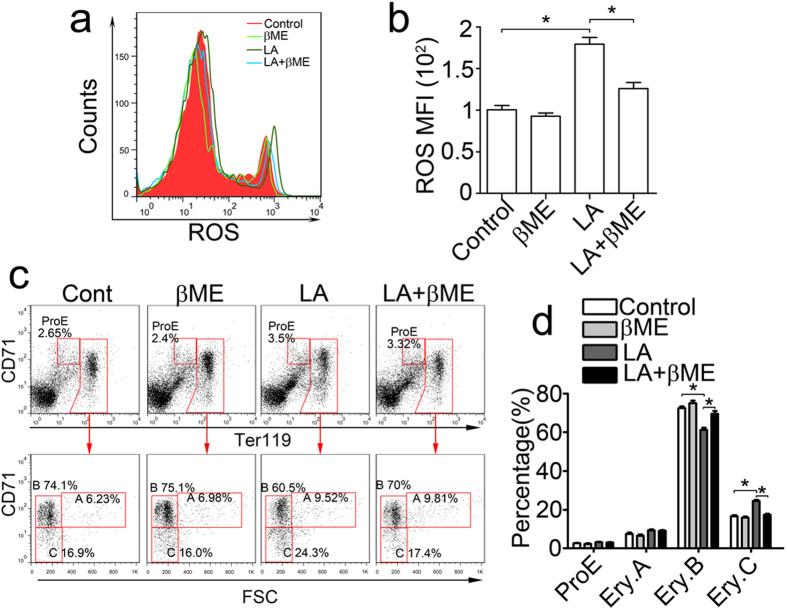
Lactic acid-enhanced erythropoiesis of mouse BM cells was inhibited by antioxidants. (**a**,**b**) BM cells stimulated with 5-mM lactic acid or 50-μM β-ME for 6 hours were analysed via flow cytometry for ROS production using DCFH-DA probes. (**a**) β-ME treatment inhibited the increased ROS production of BM cells in the lactic acid-treated group. The red curve indicates cells treated with the medium, the light green curve indicates cells treated with β-ME, the dark green curve indicates cells treated only with lactic acid, and the blue curve indicates cells treated with both lactic acid and β-ME. (**b**) MFI analyses are shown. The data represent the mean ± S.E.M. *P < 0.05. (**c**,**d**) Erythroid differentiation of BM cells promoted by lactic acid was inhibited by β-ME. (**c**) The cells were stained with CD71 and Ter119 antibodies and analysed via flow cytometry 2 days after 5-mM lactic acid and 50-μM β-ME stimulation. (**d**) The percentage profile of the populations in panel c. The data represent the mean ± S.E.M. *P < 0.05.

Although enhanced cellular ROS levels were established in lactic acid-treated BM cells and K562 cells, it is unclear how ROS regulates lactic acid-induced erythroid differentiation. 2-mercaptoethanol (β-ME) was used to evaluate the function of ROS by reducing ROS accumulation in BM cells. Flow cytometry showed that the increased ROS MFI in the lactic acid group was reduced to near normal levels in the β-ME-treated group, indicating that β-ME reduces lactate-induced ROS accumulation (Fig. 5a,b). An analysis of erythroid differentiation using flow cytometry demonstrated that the increase in the Ery.C population induced by lactic acid reduced to normal levels 2 days after β-ME treatment (Fig. 5c,d). These results indicated that ROS played an important role for the lactic acid-induced erythropoiesis of BM cells and that this process can be inhibited by the pretreatment with an antioxidant.

**Figure 6:**
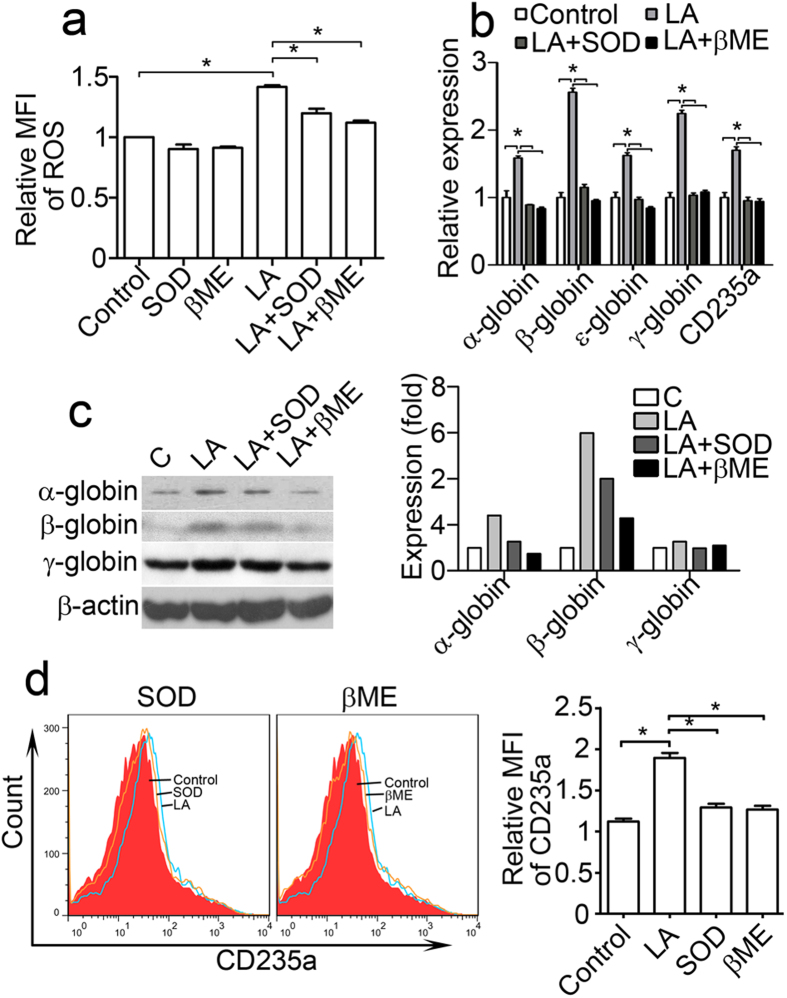
Antioxidants inhibit K562 cell erythroid differentiation enhanced by lactic acid. (**a**–**d**) K562 cells were treated with combinations of 10-mM lactic acid, 50-μM β-ME, and 50 U SOD. (**a**) K562 cells were analysed via flow cytometry for ROS production 6 hours after lactic acid, SOD and β-ME treatments. The relative mean fluorescence intensity (MFI) of ROS was calculated relative to the values of the normal group. *P < 0.05. (**b**) Real time RT-PCR was used for haemoglobin and CD235a gene expression analyses at 72 hours. mRNA levels were normalized to the expression of β-actin mRNA. *P < 0.05. (**c**,**d**) Antioxidants (SOD and β-ME) blocked the K562 erythroid differentiation promoted by 10-mM lactic acid *in vitro*. (**c**) Haemoglobin levels in the K562 cells were detected via immunoblotting 72 hours after lactic acid and antioxidant treatment. The bands were quantified using Quantity One software, and the β-actin bands were used for normalization. (**d**) Flow cytometry was used to detect the CD235a expression levels in the K562 cells 72 hours after lactic acid and antioxidant treatment. The relative MFI of CD235a were calculated normalized to the values of non stained cells. The data represent the mean ± S.E.M. *P < 0.05.

We also assessed the function of ROS on the lactate-induced differentiation of K562 cells. After antioxidant treatment with SOD or β-ME, ROS levels were markedly decreased in lactic acid-treated K562 cells, but no obvious effect on the control group (Fig. 6a). The α-, β-, γ- and ε-haemoglobin mRNA levels also decreased in SOD- and β-ME-pretreated K562 cells compared with lactic acid-treated group (Fig. 6b). Immunoblotting showed that β-ME and SOD suppressed α-, β- and γ-haemoglobin protein expression 3 days after lactic acid treatment (Fig. 6c). The *CD235a* mRNA levels in the K562 cells were measured via quantitative real-time PCR 3 days after lactic acid and SOD or β-ME treatment. The results showed that the elevation in the level of *CD235a* mRNA induced by lactic acid was inhibited by SOD and β-ME (Fig. 6b). Flow cytometry analysis further demonstrated that the up-regulation of CD235a expression on the surface of K562 cells mediated by lactic acid was reduced after SOD and β-ME treatment (Fig. 6d). These results suggest that lactate-induced erythroid differentiation can be offset by treatment with antioxidants, such as SOD and β-ME, and that this process is probably mediated by ROS.

### Signalling alternations following lactic acid treatment

**Figure 7:**
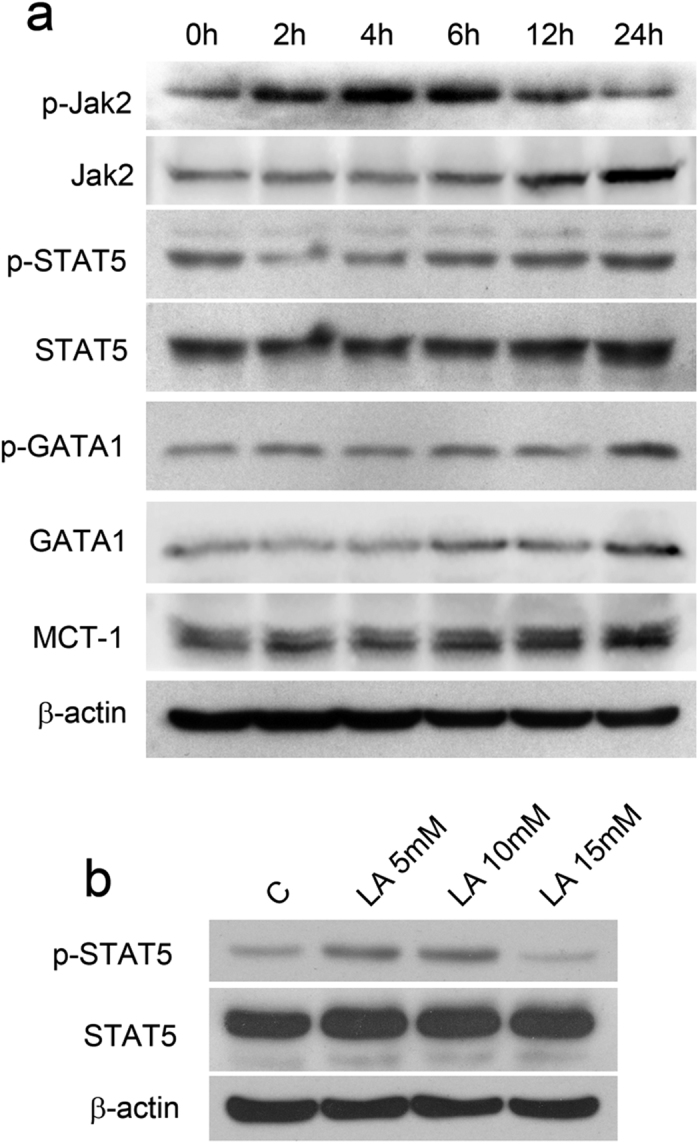
Lactic acid controls erythroid gene activation in K562 cell lines. (**a**) Expression analysis of Jak2, STAT5, GATA1 and MCT-1 in K562 whole cell extracts following stimulation with 10-mM lactic acid for the indicated times. (**b**) Expression analysis of p-STAT5 and STAT5 in K562 cell extracts 72 hours after stimulation with 5-, 10- and 15-mM lactic acid. The results are representative of three independent experiments.

To further decipher the mechanism of erythropoiesis enhanced by lactic acid, we focused on a lactate transport protein, MCT-1^30^, and previously reported erythroid-related genes, including Jak2, STAT5, and GATA1. We measured their expression in response to 10-mM lactic acid in K562 cells. Immunoblotting indicated an up-regulation of MCT-1 levels at 24 h, suggesting the increased transport of lactic acid to the cytoplasm (Fig. 7a). p-Jak2 levels increased by nearly 4.8-fold in the first 4 hours and then decreased to normal levels at 24 hours. Total Jak2 expression was increased at 24 hours by 5-fold. p-STAT5 levels initially decreased and then started to rise after Jak2 was phosphorylated and activated, with an approximate 2-fold increase. We also detected the total and p-STAT5 levels at 72 hours after various doses of lactic acid (Fig. 7b). No obvious increases were found in the total STAT5 levels, however, p-STAT5 increased by 2.5-fold and 3-fold, respectively in the 5- and 10-mM groups (Fig. 7b). Western blot analysis also showed an increase in GATA1 expression and in its phosphorylation level 24 hours after lactic acid treatment compared with the levels at 0 hours (Fig. 7a). These data indicate that the observed lactic acid-induced erythroid differentiation may be regulated by the activation of several of these key erythroid-related signalling proteins.

## Discussion

Lactic acid is a small molecule that is utilized as an energy source by cells, but it also plays other essential roles with regard to cellular functionality. In this study, we found that lactic acid was able to differentiate the human leukaemia cell line K562 towards erythroid cell lineages. Lactic acid treatment increased haemoglobin mRNA and protein expression levels, with a concomitant increase in GPA expression. We also found that lactic acid promoted the terminal differentiation of BM-derived HSCs towards the erythroid lineage *in vivo*. The progression of events that results in this lineage commitment increases the number of RBCs and haemoglobin levels while also enhancing the BFU-E colony formation ability of CD34^+^/CD117^−^ haematopoietic progenitors stimulated by lactic acid treatment *in vitro*. These studies indicate the role played by lactic acid in erythroid differentiation and may provide a possible mechanism for the relation between high lactic acid levels and erythrocytosis that has been observed in some tumour models^18^,^19^,^31^.

Accumulating evidences have shown that erythropoiesis responds to environmental stresses such as hypoxia and low temperature^32^,^33^,^34^,^35^. Cell metabolism alters significantly and produces very different metabolic produces, such as lactic acid, when HSCs adopt to environmental stresses. Though both of erythropoiesis and lactic acid increase during hypoxia, we have known little about the effects of lactic acid to erythropoiesis. A previous study reported that lactic acid modulates serum EPO levels after exposure to hypoxia or cobalt^20^, and our results also showed an increase in plasma EPO at 24 or 48 hours after *in vivo* lactic acid administration (Supplementary Figure 2). Nevertheless, because of the lack of any detectable erythropoietin *in vitro*, erythropoietin cannot explain the lactate-induced erythroid differentiation of K562 cells and HSCs, which suggests that there is another signal pathway responsible for the enhanced erythropoiesis. It has been reported that lactic acid can modulate cellular metabolism and function by altering the cell’s oxidative status, and it is through ROS that lactic acid regulates myogenesis, enhances cell-cycle withdrawal, and initiates early differentiation while simultaneously delaying late differentiation in a time- and dose-dependent manner in C2C12 myoblasts^36^. Lactic acid regulates germ cell function in male rats by stimulating ROS production. In this setting, ROS acts as a second messenger that regulates gene expression while modulating pivotal aspects of specific signal transduction pathways^37^. In agreement with these findings, we demonstrated that lactic acid increases oxidative stress in BM and K562 cells. ROS production increased as the concentration of lactic acid increased, and the effect of lactic acid on ROS production was detectable as early as 15 minutes after treatment and continued for more than 6 hours. The antioxidants SOD and β-ME impaired the production of ROS stimulated by lactic acid. Flow cytometry showed that these antioxidants reversed erythroid differentiation, decreased GPA expression in K562 cells and down-regulated haemoglobin expression, indicating that ROS play a role in lactate-induced erythroid differentiation. Although the role of ROS in erythropoiesis is controversial, some reports have shown that ROS induced by benzene or hydroquinone inhibit erythroid differentiation in HD3 chicken erythroblast cells and K562 cells^38^,^39^. Other studies have shown that increased levels of ROS in the early stage of erythroblast development sensitize them to differentiate towards mature blood cells^28^,^40^. In addition to affecting erythroid differentiation, lactic acid may also contribute to cellular process such as Th17 cell differentiation, where the role of ROS as second messengers is equally essential^41^.

A high rate of lactic acid production is a characteristic feature of some cancers because of enhanced glycolysis in tumour tissues, which significantly influences the body’s normal functions^16^. It has been reported that lactic acid drives endothelial cell migration and tube formation by triggering the phosphorylation/degradation of IκBα and by stimulating the NF-κB/IL-8 (CXCL8) pathway, promoting tumour angiogenesis^42^. During DC differentiation, the addition of lactic acid induces a phenotype comparable to tumour-associated dendritic cells, which may contribute to the tumour escape mechanism^43^. Lactic acid also suppresses the proliferation and cytokine production of human cytotoxic T lymphocytes (CTLs) by up to 95% and leads to a 50% decrease in cytotoxic activity^44^. These previous studies suggest that lactic acid may have the ability to promote tumour growth by affecting the immune system or angiogenesis. We have demonstrated that lactic acid (5 mM) can promote the erythroid differentiation of HSCs and that high doses of lactic acid (10 mM) increased the apoptosis of HSC cells (Supplementary Figure 1). Therefore, we speculated that tumour cells may utilize lactic acid-enhanced erythropoiesis to produce more red blood cells for increased oxygen transport.

The progressive activation of signalling pathways along with transcriptional factors plays an important role in regulating cell differentiation, proliferation and death. In erythroid differentiation, the main factors include Jak2, STAT5 and GATA1^45^,^46^. Upon activation of cytokine receptors, such as the EPO receptor (EPOR), Jak2 proteins are autophosphorylated, and their catalytic activities are controlled by the phosphorylation of Y1007^47^,^48^. A Jak2 deficiency results in the absence of definitive erythropoiesis, as demonstrated by BFU-E and CFU-E colony formation^45^. STAT5 is phosphorylated after Jak2, and receptor activation is followed by its translocation into the nucleus and the activation of the promoters of downstream genes^46^. A deficiency of STAT5 has been shown to lead to a failure of differentiation, although erythroblast numbers were found to be dramatically increased^49^. The transcription factor GATA1 is essential for normal erythropoiesis, and its activation is required for organizing the haematopoietic lineage commitment in forming myeloerythroid lineages^32^,^50^,^51^. Studies have also shown that GATA1 regulates the expression level of the β-globin locus in a time-dependent manner by binding the enhancer^52^,^53^. In the present study, we found that lactic acid increased p-Jak2 levels 4 hours after treatment. The STAT5 phosphorylation level began to increase after Jak2 was activated and was maintained at a high level in the 5- and 10-mM groups at 72 hours. The total and P-GATA1 protein levels increased at 24 hours after 10-mM lactic acid treatment. Our findings suggest that lactic acid activates erythroid differentiation, possibly through these erythroid transcriptional factors and signalling pathways. Our findings are in line with those of previous studies that have described the function of GATA1, Jak2 and STAT5 in erythropoiesis^45^,^46^. However, the detailed molecular mechanism underlying this process remains largely unknown and requires further investigation in our future studies.

In conclusion, we have demonstrated a new role of lactic acid in erythroid differentiation. This process may be mediated by ROS and erythroid transcription factors. These findings expand on and improve our understanding of the role of lactic acid in cellular metabolism and physiological functions.

## Materials and Methods

### Animals and ***in vivo*** lactic acid treatment

All animal experiments were performed using female Balb/c mice (6- to 8-week-old adult mice, Vital River Company, Beijing, China). The mice were housed in pathogen-free facilities according to the standard institutional guidelines of the Gene-Engineered Mouse Core Facility at the State Key Laboratory of Biotherapy at Sichuan University. To establish the lactic acid-treated mouse model, 12 mice were divided into 4 groups and administered a single intraperitoneal (i.p.) injection of lactic acid at 1.25, 0.63, 0.31 or 0 mmol/kg (Groups = 4, N = 3) in a total volume of 1 ml PBS. Animals were sacrificed 48 hours later. All animal experimental procedures were performed and approved in accordance with the guidelines for animal research of Institutional Animal Care and Use Committee of the State Key Laboratory of Biotherapy at Sichuan University.

### Haematocrit, red blood cells count and haemoglobin levels in plasma

Whole blood (50 μL) was collected from mice via the retro-orbital plexuses into Eppendorf tubes containing EDTA. Approximately 20 μl of whole blood was used to test haematocrit levels, red blood cell counts and plasma haemoglobin levels using a Nihon Kohden MEK 6318 K haematology analyser (Nihon Kohden, Tokyo, Japan).

### EPO and lactic acid level measurements

Whole blood (100 μl) collection was performed as described as above. The plasma was separated by centrifugation at 4,000 rpm for 15 min and stored at −80 °C until assayed. EPO levels were measured using a Mouse Erythropoietin/EPO ELISA Kit (LifeSpan BioSciences) according to the manufacturer’s instructions. Lactic acid was assayed using a L-lactate Assay Kit I (Eton Bioscience) according to the manufacturer’s instructions.

### Cell culture and colony formation assays

Bone marrow cells were freshly isolated by flushing the tibia and femur of the sacrificed mice and were resuspended in RPMI 1640 medium (Hyclone). The cell suspensions were filtered through a 40 μm cell strainer (Millipore) to obtain a uniform single-cell suspension, and then cells were counted. The cells were cultured in RPMI 1640 medium supplemented 10% FBS at 37 °C in 5% CO_2_. K562 cells lines were purchased from the American Type Culture Collection (ATCC, USA) and cultured in RPMI 1640 medium with 10% FBS at 37 °C in 5% CO_2_ in air. For the colony formation assays, whole BM cells were resuspended in PBS and stained with CD34 or CD117 antibody-coated microbeads (Miltenyi Biotec, Bergisch Gladbach, Germany). The CD34^+^/CD117^−^ cells were collected, counted and seeded onto 35-mm plastic culture dishes with methylcellulose media (Stemcell Technologies, Vancouver, Canada). The cells were cultured at 37 °C in 5% CO_2_ in air at high humidity for 10 days. On day 10, the number of erythroid burst-forming units (BFU-Es) was counted.

### Flow cytometry

Cells were collected and resuspended in PBS with specific fluorescently labelled antibodies at 4 °C for 30 minutes in the dark. Cells stained with an isotype control antibody were used as controls. Subsequently, the cells were washed and finally resuspended in PBS in the dark for analysis using a BD FACS Calibur system, as previously described. A FITC-conjugated monoclonal antibody specific to mouse CD71, a PE-conjugated monoclonal antibody specific to mouse TER-119 and the corresponding isotype control antibodies were all purchased from BD Pharmingen (San Diego, CA). Primary antibodies were used at a 1:100 dilution. K562 cells were stained with a PE-conjugated monoclonal antibody specific to human CD235a (eBioscience, San Diego, CA). The primary antibodies were used at a concentration of 0.5 μg/ml. For the apoptosis assay, the cells were stained with Annexin V-FITC (BD Pharmingen, CA) and PI (BD Pharmingen, CA) following the manufacturer’s protocol.

### RNA isolation and reverse transcriptase-PCR (RT-PCR)

Total RNA was isolated from K562 cells using TRIzol reagent (Tiangen Biotech Co. LTD., China). RNA purity and concentration were measured via spectrophotometry (Thermo Fisher Scientific), and RNA integrity was verified via gel-electrophoresis. Single-stranded cDNA was generated from total RNA (1 μg) using random hexamers from a PrimeScript RT Reagent Kit and gDNA Eraser (Takara Biochemicals, Kyoto, Japan) in a volume of 20 μl. The cDNA was amplified using SYBR Green Supermix (Bio-Rad, Hercules, CA) in a thermal cycler (Bio-Rad, Hercules, CA) with specific primer pairs for human β-actin, α-haemoglobin, β-haemoglobin, γ-haemoglobin, ε-haemoglobin, and CD235a. After an initial denaturation at 90 °C for 2 min, the samples were amplified for 40 cycles using the following PCR cycle conditions: 95 °C for 5 s and 60 °C for 30 s each. The PCR primers used in the experiment are shown in Table 1 54.

### Western blotting

Western blot analysis was performed as previously described^55^. Briefly, cells were collected and lysed in RIPA buffer (Beyotime Biotechnology, China) and protein concentration was quantified using a BCA assay (Thermo Fisher Scientific, USA). Equivalent amounts of protein extracts were separated via SDS-PAGE electrophoresis and then transferred onto PVDF membranes (0.2 μm, Millipore, USA). The membranes were blocked with 5% skim milk in TBS containing 0.1% (v/v) Tween-20 (TBST) at room temperature for 1 h and probed at 4 °C overnight with primary antibodies. β-actin and γ-haemoglobin antibodies were purchased from Santa Cruz Biotechnology (Santa Cruz, USA); P–Jak2, P-STAT5, Jak2 and STAT5 antibodies were obtained from Cell Signaling Technology; α-haemoglobin, β-haemoglobin, P-GATA1 and GATA1 antibodies were purchased from Abcam. Following 4 washes with TBST, the membranes were incubated with horseradish peroxidase (HRP)-conjugated secondary antibodies (1:10000, in blocking buffer) for 1 hour at room temperature. Bands were detected using enhanced chemiluminescence detection reagents (Millipore, USA). The bands were quantified using the Quantity One software, and the β-actin bands were used for normalization.

### ROS and hydrogen peroxide measurements

Cellular hydrogen peroxide (H_2_O_2_) generation was measured as previously described^29^. Briefly, cells were cultured in 1640 medium supplemented with 10-mM mercaptosuccinic acid (a glutathione peroxidase inhibitor, Sigma-Aldrich, China) and 30-mM 3-amino-1,2,4-triazole (a catalase inhibitor, Sigma-Aldrich, China) for 30 min. Then, the cells were incubated with or without lactic acid and harvested 3, 5, 10 or 15 minutes later. Following two washes with PBS, the cells were lysed in a lysis buffer and centrifuged to obtain the supernatant. H_2_O_2_ levels were measured using a Hydrogen Peroxide Assay Kit (Bio-Assay System, Hayward, CA, USA) at 570 nm. Cellular ROS production was measured using a dichlorodihydrofluorescein-diacetate (DCFH-DA) probe. Approximately 1 × 10^6^ cells were cultured in the presence or absence of lactic acid for 6 hours, washed twice with sterile HBSS and resuspended in RPMI 1640 medium without FBS. A final concentration of 10 μM DCFH-DA was added to the cells, and they were then incubated for 30 min. Finally, the cells were harvested, washed and suspended in PBS. Fluorescence intensity measurements were carried out using flow cytometry, and cells incubated without lactic acid served as the control.

### Statistical analyses

The results are expressed as the mean ± SEM. Student’s t tests and one-way ANOVAs (followed by Tukey’s post-hoc tests) were used. P < 0.05 (*) were considered significant in the experiments.

## Additional Information

**How to cite this article**: Luo, S.-T. *et al*. The Promotion of Erythropoiesis via the Regulation of Reactive Oxygen Species by Lactic Acid. *Sci. Rep.* **7**, 38105; doi: 10.1038/srep38105 (2017).

**Publisher's note:** Springer Nature remains neutral with regard to jurisdictional claims in published maps and institutional affiliations.

## Supplementary Material

Supplementary Information

## Figures and Tables

**Table 1:** Quantitative real-time RT-PCR primers used for the K562 cells.

Gene	Forward Primer (5′-3′)	Reverse Primer (3′-5′)
β-actin	CTGGCACCACACCTTCTACA	AGCACAGCCTGGATAGCAAC
α-globin	GGTCAACTTCAAGCTCCTAAGC	GCTCACAGAAGCCAGGAACTTG
β-globin	GTCTACCCTTGGACCCAGAGGTTC	TGAGCCAGGCCATCACTAAAG
γ-globin	GCAGCTTGTCACAGTGCAGTTC	TGGCAAGAAGGTGCTGACTTC
ε-globin	CAGCTGCAATCACTAGCAAGC	AGACGACAGGTTTCCAAAGC
CD235a	GGCTGGTGTTATTGGAACGATC	GAGGTTTTACATCAGATGGGCTTT
