# Supplementary material for: The Promotion of Erythropoiesis via the Regulation of Reactive Oxygen Species by Lactic Acid
Source: Sci Rep. 2017 Feb 6;7:38105. doi: 10.1038/srep38105 (PMC5292721; doi:10.1038/srep38105)
Supplement: Supplementary Information [file srep38105-s1.pdf]

Supplementary Information

The Promotion of Erythropoiesis via the Regulation of Reactive Oxygen Species by Lactic Acid

Shun-Tao Luo[1*], Dong-Mei Zhang[2*], Qing Qin[3*], Lian Lu[1], Min Luo[1], Fu-Chun Guo[1], Hua-Shan Shi[4], Li Jiang[5], Bin Shao[1], Meng Li[1], Han-Shuo Yang[1#], Yu-Quan Wei[1#]

[1]State Key Laboratory of Biotherapy and Cancer Center, West China Hospital, Sichuan University, and Collaborative Innovation Center for Biotherapy, Chengdu, 610064, China.

[2]Center of Reproductive Medicine，Department of Gynecology and Obstetrics, State Key Laboratory of Biotherapy, West China Second Hospital, Sichuan University, and Collaborative Innovation Center for Biotherapy, Chengdu, China.

[3]Department of Oncology, Chengdu Shang Jin Nan Fu Hospital, Chengdu, Sichuan 610041, China.

[4]State Key Laboratory of Biotherapy and Cancer Center, West China Hospital, Sichuan University, and Collaborative Innovation Center for Biotherapy, and Head and Neck Oncology Department of Cancer Center, West China Hospital, Chengdu, 610064, China.

[5]West China Hospital, West China Medical School, Sichuan University, Chengdu, 610064, China.

*These authors contributed equally to this work

[#]Whom corresponding to: yqwei@scu.edu.cn or yhansh@scu.edu.cn

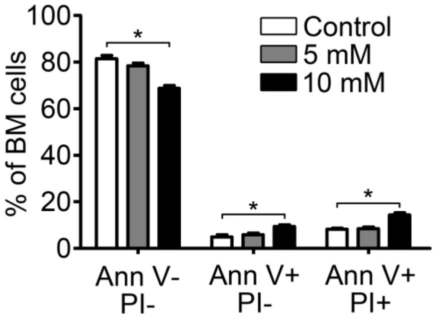

Supplementary Figure S1. High doses of lactic acid induced apoptosis of BM cells cultured *in vitro*. Apoptosis of BM cells cultured with lactic acid or medium for 48 hours and assessed by flow cytometry of PI and annexin V (Ann V) staining. The data represent the mean ± S.E.M.(n=3). *P<0.05.

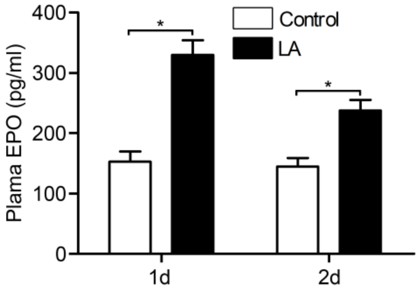

Supplementary Figure S2. Plasma EPO levels increased in the lactic acid-treated mice. Increased plasma EPO levels relative to the control levels were detected in the mice at day 1 and day 2 in the 1.25 mmol/kg LA group. The data represent the mean ± S.E.M., *P<0.05.

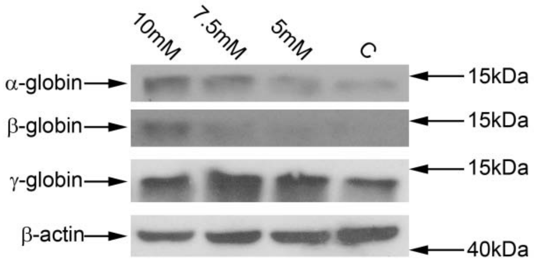

Supplementary Figure S3. Lactic acid induces haemoglobin expression in K562 cells Haemoglobin expression in K562 cells at 72 hours after treatment with 5-, 7.5- or 10-mM lactic acid. These cropped blots are used in the main figure (Figure 1) and these full-length blots are included in the supplementary figure

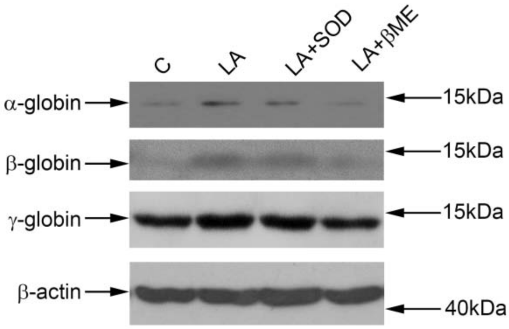

Supplementary Figure S4. Haemoglobin expression is inhibited by antioxidants in lactic acid-treated K562 cells. Haemoglobin levels in the K562 cells were detected via immunoblotting 72 hours after lactic acid and antioxidant treatment. These cropped blots are used in the main figure (Figure 6) and these full-length blots are included in the supplementary figure

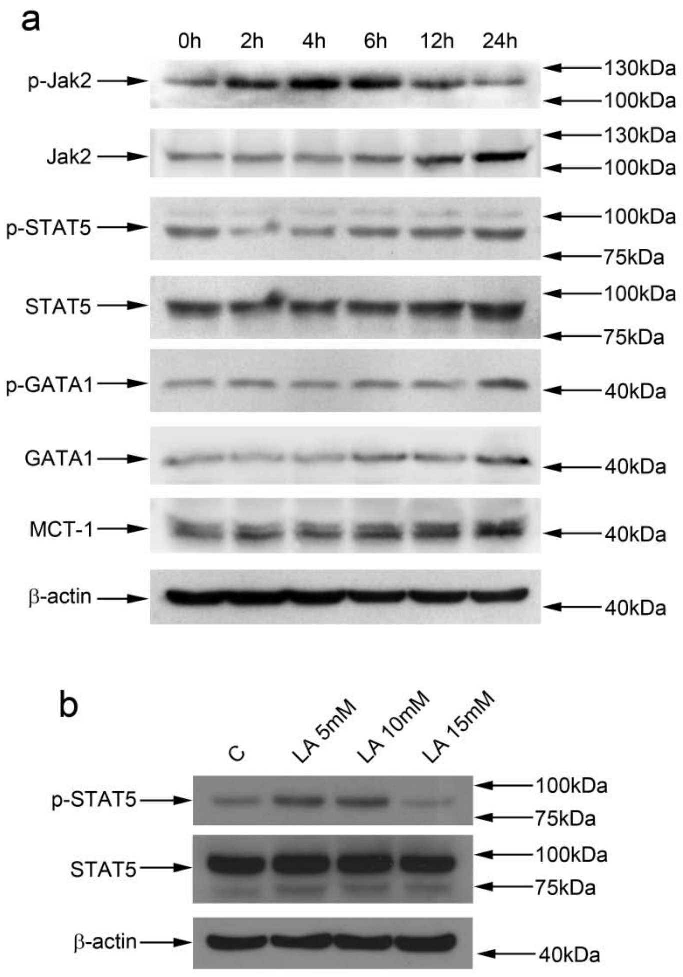

Supplementary Figure S5. Lactic acid controls erythroid gene activation in K562 cell lines. (a) Expression analysis of Jak2, STAT5, GATA1 and MCT-1 in K562 whole cell extracts following stimulation with 10-mM lactic acid for the indicated times. (b) Expression analysis of p-STAT5 and STAT5 in K562 cell extracts 72 hours after stimulation with 5-, 10-and 15-mM lactic acid. These cropped blots are used in the main figure (Figure 7) and these full-length blots are included in the supplementary figure
